# 7-Amino-4-hy­droxy-4-trifluoro­methyl-3,4-dihydro­quinolin-2(1*H*)-one

**DOI:** 10.1107/S1600536811035471

**Published:** 2011-09-14

**Authors:** Yuan Qin, Haitao Xi, Liang Chen, Xiaoqiang Sun

**Affiliations:** a Key Laboratory of Fine Chemical Engineering, Changzhou University, Changzhou 213164, Jiangsu, People’s Republic of China

## Abstract

The title compound, C_10_H_9_F_3_N_2_O_2_, was prepared by the reaction of *m*-phenyl­enediamine and ethyl 4,4,4-trifluoro­acetoacetate. In the crystal, inter­molecular C—H⋯ F, N—H⋯F, O—H⋯N and N—H⋯O inter­actions contribute to the crystal packing.

## Related literature

For general background to quinolino­nes, see: Chilin *et al.* (1991 ▶); Oeveren *et al.* (2006 ▶). For related structures, see: Oeveren *et al.* (2007 ▶). For ring conformation analysis, see Cremer & Pople (1975 ▶).
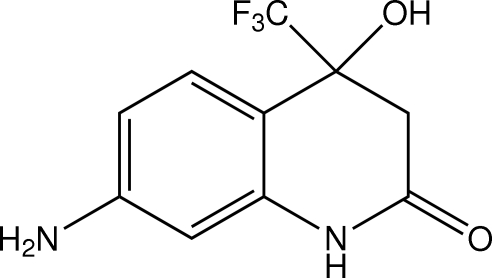


## Experimental

### 

#### Crystal data

C_10_H_9_F_3_N_2_O_2_
*M* *_r_* = 246.19
Monoclinic, 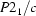

*a* = 8.6770 (9) Å
*b* = 10.0816 (11) Å
*c* = 11.6293 (12) Å
β = 95.747 (2)°
*V* = 1012.20 (18) Å^3^
*Z* = 4
Mo *K*α radiation
μ = 0.15 mm^−1^
*T* = 296 K
0.20 × 0.18 × 0.15 mm

#### Data collection

Bruker SMART APEX CCD diffractometer
Absorption correction: multi-scan (*SADABS*; Bruker, 2000 ▶) *T*_min_ = 0.971, *T*_max_ = 0.978
6400 measured reflections
2283 independent reflections
2021 reflections with *I* > 2σ(*I*)
*R*_int_ = 0.028

#### Refinement

*R*[*F* ^2^ > 2σ(*F* ^2^)] = 0.040
*wR*(*F* ^2^) = 0.112
*S* = 1.06
2283 reflections
170 parameters
H atoms treated by a mixture of independent and constrained refinement
Δρ_max_ = 0.33 e Å^−3^
Δρ_min_ = −0.28 e Å^−3^

### 

Data collection: *SMART* (Bruker, 2000 ▶); cell refinement: *SAINT* (Bruker, 2000 ▶); data reduction: *SAINT*; program(s) used to solve structure: *SHELXS97* (Sheldrick, 2008 ▶); program(s) used to refine structure: *SHELXL97* (Sheldrick, 2008 ▶); molecular graphics: *SHELXTL* (Sheldrick, 2008 ▶); software used to prepare material for publication: *SHELXTL*. 

## Supplementary Material

Crystal structure: contains datablock(s) I, global. DOI: 10.1107/S1600536811035471/mw2019sup1.cif

Structure factors: contains datablock(s) I. DOI: 10.1107/S1600536811035471/mw2019Isup2.hkl

Supplementary material file. DOI: 10.1107/S1600536811035471/mw2019Isup3.cml

Additional supplementary materials:  crystallographic information; 3D view; checkCIF report

## Figures and Tables

**Table 1:** Hydrogen-bond geometry (Å, °)

*D*—H⋯*A*	*D*—H	H⋯*A*	*D*⋯*A*	*D*—H⋯*A*
N1—H1*A*⋯O1^i^	0.91 (2)	2.17 (2)	3.0409 (16)	160.0 (18)
N2—H1*B*⋯O2^ii^	0.92 (2)	1.99 (2)	2.9075 (15)	175.3 (18)
O1—H1*C*⋯N1^iii^	0.82 (2)	2.02 (2)	2.8280 (16)	168 (2)
N1—H2*A*⋯F3^iv^	0.87 (2)	2.41 (2)	3.1124 (16)	138.5 (17)
C4—H4⋯F2^v^	0.93	2.31	3.1846 (16)	156

Symmetry codes: (i) 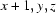; (ii) 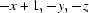; (iii) 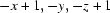; (iv) 
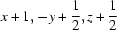
; (v) 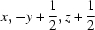.

**Fig. 1.**
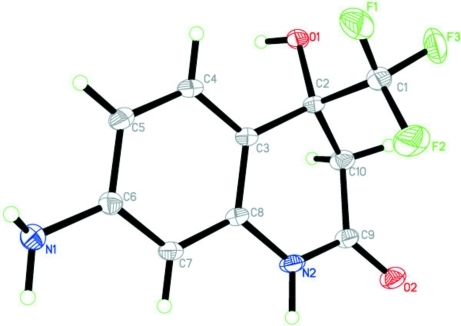
A perspective view of the title compound . Displacement ellipsoids are drawn at the 30% probability level.

**Fig. 2.**
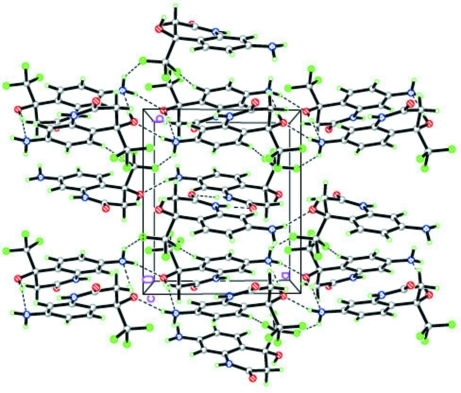
Crystal packing viewed down the crystallographic c axis showing the hydrogen bonding interactions.

## supplementary crystallographic information

### Comment

Owing to applications in biology and medicine, for example as orally available tissue-selective androgen receptor modulators, quinolinone and its derivatives have been extensively studied (Chilin, *et al.*, 1991; Oeveren, *et al.*, 2006). During a preparation of 7-amino-4-trifluoromethylquinolinone according to the published procedure (Oeveren, *et al.*, 2007), 7-amino-4-hydroxy-4-(trifluoromethyl)-3,4-dihydroquinolin-2(1*H*)-one was unexpectedly obtained when a lower reaction temperature than that in the literature was employed. The product was characterized by ^1^H NMR and the structure confirmed by the present determination (Fig. 1).

In the title compound, the six-membered ring (C2, C3, N2, C8, C9, C10) adopts a non-classical conformation which is chracterized by the Cremer-Pople puckering parameters: Amplitude (Q) = 0.3577 (15) Å , Θ = 117.9 (2) ° , Φ = 44.6 (3) °) (Cremer & Pople 1975). Intermolecular C–H···F, N–H···F, O–H···N, O–H···O and N–H···O interactions link the molecules and contribute to the crystal packing (Fig. 2).

### Experimental

A mixture of *m*-phenylenediamine (100 mmol, 10.8 g) and ethyl 4,4,4-trifluoroacetoacetate (100 mmol, 15.2 mL) was heated at 353 K for 48 h. The cooled reaction mixture was suspended in 20 mL of methanol and the undissolved solid was collected. The pure product crystallized from ethanol to afford a white solid (80% yied, m.p. 501 K). ^1^H NMR (500 MHz, [D6] DMSO): *δ* 10.13 (bs, 1 H, NH), 7.16(d, *J* = 7.5 Hz, 1 H, 4-quin), 6.59(s, 1 H, OH), 6.23(d, *J* = 7.5 Hz, 1 H, 5-quin ), 6.10(d, *J* = 1.5 Hz, 1 H, 7-quin), 5.39(bs, 2 H, NH_2_ ), 2.83(dd, *J* = 1.6, 4.9 Hz, 2 H, 2-quin). Single crystals suitable for X-ray diffraction were obtained by evaporation of an ethanol solution.

### Refinement

All H atoms bonded to C atoms were placed in geometrically idealized positions and constrained to ride on their parent atoms with C—H distances of 0.93–0.97 Å, and with *U*_iso_(H) = 1.2*U*_eq_(C). The other H atoms bonded to N and O atom were found in the difference maps and were refined independently.

### Crystal data

**Table:**

C_10_H_9_F_3_N_2_O_2_	*F*(000) = 504
*M**_r_* = 246.19	*D*_x_ = 1.616 Mg m^−^^3^
Monoclinic, *P*2_1_/*c*	Mo *K*α radiation, λ = 0.71073 Å
Hall symbol: -P 2ybc	Cell parameters from 4544 reflections
*a* = 8.6770 (9) Å	θ = 2.4–30.4°
*b* = 10.0816 (11) Å	µ = 0.15 mm^−^^1^
*c* = 11.6293 (12) Å	*T* = 296 K
β = 95.747 (2)°	Prism, yellow
*V* = 1012.20 (18) Å^3^	0.20 × 0.18 × 0.15 mm
*Z* = 4	

### Data collection

**Table:**

Bruker SMART APEX CCD diffractometer	2283 independent reflections
Radiation source: fine-focus sealed tube	2021 reflections with *I* > 2σ(*I*)
graphite	*R*_int_ = 0.028
φ and ω scans	θ_max_ = 27.5°, θ_min_ = 2.4°
Absorption correction: multi-scan (*SADABS*; Bruker, 2000)	*h* = −11→10
*T*_min_ = 0.971, *T*_max_ = 0.978	*k* = −12→8
6400 measured reflections	*l* = −15→15

### Refinement

**Table:**

Refinement on *F*^2^	Primary atom site location: structure-invariant direct methods
Least-squares matrix: full	Secondary atom site location: difference Fourier map
*R*[*F*^2^ > 2σ(*F*^2^)] = 0.040	Hydrogen site location: inferred from neighbouring sites
*wR*(*F*^2^) = 0.112	H atoms treated by a mixture of independent and constrained refinement
*S* = 1.06	*w* = 1/[σ^2^(*F*_o_^2^) + (0.0568*P*)^2^ + 0.3518*P*] where *P* = (*F*_o_^2^ + 2*F*_c_^2^)/3
2283 reflections	(Δ/σ)_max_ < 0.001
170 parameters	Δρ_max_ = 0.33 e Å^−^^3^
0 restraints	Δρ_min_ = −0.28 e Å^−^^3^

### Special details

**Table:**

Geometry. All esds (except the esd in the dihedral angle between two l.s. planes) are estimated using the full covariance matrix. The cell esds are taken into account individually in the estimation of esds in distances, angles and torsion angles; correlations between esds in cell parameters are only used when they are defined by crystal symmetry. An approximate (isotropic) treatment of cell esds is used for estimating esds involving l.s. planes.
Refinement. Refinement of F^2^ against ALL reflections. The weighted R-factor wR and goodness of fit S are based on F^2^, conventional R-factors R are based on F, with F set to zero for negative F^2^. The threshold expression of F^2^ > 2sigma(F^2^) is used only for calculating R-factors(gt) etc. and is not relevant to the choice of reflections for refinement. R-factors based on F^2^ are statistically about twice as large as those based on F, and R- factors based on ALL data will be even larger.

### Fractional atomic coordinates and isotropic or equivalent isotropic displacement parameters (Å^2^)

**Table:**

	*x*	*y*	*z*	*U*_iso_*/*U*_eq_	
C1	0.12400 (17)	0.21289 (16)	0.23013 (12)	0.0372 (3)	
C2	0.19252 (14)	0.08079 (13)	0.27561 (10)	0.0268 (3)	
C3	0.35833 (14)	0.10123 (13)	0.32715 (10)	0.0258 (3)	
C4	0.39150 (15)	0.15288 (14)	0.43763 (11)	0.0313 (3)	
H4	0.3104	0.1748	0.4807	0.038*	
C5	0.54182 (16)	0.17229 (14)	0.48484 (11)	0.0316 (3)	
H5	0.5613	0.2075	0.5588	0.038*	
C6	0.66507 (14)	0.13917 (13)	0.42169 (11)	0.0272 (3)	
C7	0.63442 (14)	0.08873 (14)	0.31075 (10)	0.0280 (3)	
H7	0.7158	0.0669	0.2679	0.034*	
C8	0.48175 (14)	0.07080 (13)	0.26335 (10)	0.0254 (3)	
C9	0.31363 (15)	−0.01002 (14)	0.09840 (11)	0.0292 (3)	
C10	0.18645 (15)	−0.02086 (15)	0.17766 (12)	0.0340 (3)	
H10A	0.1903	−0.1089	0.2114	0.041*	
H10B	0.0875	−0.0122	0.1314	0.041*	
F1	0.13125 (13)	0.30710 (10)	0.31014 (9)	0.0552 (3)	
F2	0.19747 (15)	0.25830 (12)	0.14294 (10)	0.0652 (4)	
F3	−0.02624 (11)	0.20090 (12)	0.19086 (9)	0.0572 (3)	
N1	0.81794 (14)	0.15133 (14)	0.47467 (11)	0.0333 (3)	
N2	0.45399 (13)	0.02567 (12)	0.14878 (9)	0.0309 (3)	
O1	0.09075 (11)	0.04442 (11)	0.35806 (8)	0.0334 (3)	
O2	0.29139 (12)	−0.03941 (11)	−0.00459 (8)	0.0377 (3)	
H1B	0.532 (2)	0.026 (2)	0.1011 (16)	0.048 (5)*	
H1A	0.893 (3)	0.136 (2)	0.4276 (17)	0.053 (5)*	
H2A	0.833 (2)	0.225 (2)	0.5140 (17)	0.049 (5)*	
H1C	0.129 (2)	−0.013 (2)	0.4017 (17)	0.052 (6)*	

### Atomic displacement parameters (Å^2^)

**Table:**

	*U*^11^	*U*^22^	*U*^33^	*U*^12^	*U*^13^	*U*^23^
C1	0.0338 (7)	0.0466 (8)	0.0327 (7)	0.0068 (6)	0.0103 (6)	0.0097 (6)
C2	0.0231 (6)	0.0364 (7)	0.0223 (5)	0.0016 (5)	0.0089 (4)	0.0026 (5)
C3	0.0233 (6)	0.0314 (6)	0.0238 (6)	0.0016 (5)	0.0075 (4)	−0.0006 (5)
C4	0.0293 (6)	0.0400 (7)	0.0261 (6)	0.0047 (5)	0.0104 (5)	−0.0051 (5)
C5	0.0336 (7)	0.0381 (7)	0.0236 (6)	0.0006 (5)	0.0056 (5)	−0.0068 (5)
C6	0.0257 (6)	0.0291 (6)	0.0270 (6)	−0.0004 (5)	0.0041 (5)	0.0017 (5)
C7	0.0247 (6)	0.0346 (7)	0.0263 (6)	0.0008 (5)	0.0098 (5)	−0.0016 (5)
C8	0.0266 (6)	0.0287 (6)	0.0220 (6)	0.0002 (5)	0.0082 (4)	−0.0017 (5)
C9	0.0301 (6)	0.0326 (7)	0.0259 (6)	−0.0018 (5)	0.0078 (5)	−0.0039 (5)
C10	0.0277 (6)	0.0437 (8)	0.0316 (7)	−0.0082 (5)	0.0086 (5)	−0.0051 (6)
F1	0.0573 (6)	0.0454 (6)	0.0630 (7)	0.0190 (5)	0.0057 (5)	−0.0057 (5)
F2	0.0781 (8)	0.0630 (7)	0.0605 (7)	0.0136 (6)	0.0360 (6)	0.0336 (6)
F3	0.0394 (5)	0.0721 (7)	0.0575 (6)	0.0128 (5)	−0.0082 (4)	0.0183 (5)
N1	0.0269 (6)	0.0430 (7)	0.0301 (6)	−0.0026 (5)	0.0028 (5)	−0.0002 (5)
N2	0.0270 (5)	0.0439 (7)	0.0235 (5)	−0.0030 (5)	0.0103 (4)	−0.0072 (5)
O1	0.0246 (5)	0.0497 (6)	0.0278 (5)	0.0051 (4)	0.0116 (4)	0.0112 (4)
O2	0.0361 (5)	0.0519 (6)	0.0257 (5)	−0.0045 (4)	0.0057 (4)	−0.0085 (4)

### Geometric parameters (Å, °)

**Table:**

C1—F1	1.3267 (19)	C6—N1	1.4109 (17)
C1—F2	1.3318 (17)	C7—C8	1.3947 (17)
C1—F3	1.3429 (18)	C7—H7	0.9300
C1—C2	1.531 (2)	C8—N2	1.4057 (16)
C2—O1	1.4153 (14)	C9—O2	1.2301 (16)
C2—C3	1.5162 (17)	C9—N2	1.3465 (17)
C2—C10	1.5292 (18)	C9—C10	1.5110 (17)
C3—C4	1.3896 (17)	C10—H10A	0.9700
C3—C8	1.3966 (16)	C10—H10B	0.9700
C4—C5	1.3776 (19)	N1—H1A	0.91 (2)
C4—H4	0.9300	N1—H2A	0.87 (2)
C5—C6	1.3970 (17)	N2—H1B	0.92 (2)
C5—H5	0.9300	O1—H1C	0.82 (2)
C6—C7	1.3875 (18)		
			
F1—C1—F2	107.17 (14)	C5—C6—N1	119.03 (12)
F1—C1—F3	106.21 (12)	C6—C7—C8	120.07 (11)
F2—C1—F3	107.12 (12)	C6—C7—H7	120.0
F1—C1—C2	113.02 (12)	C8—C7—H7	120.0
F2—C1—C2	111.29 (12)	C7—C8—C3	120.67 (11)
F3—C1—C2	111.68 (13)	C7—C8—N2	118.84 (10)
O1—C2—C3	113.53 (10)	C3—C8—N2	120.46 (11)
O1—C2—C10	110.72 (11)	O2—C9—N2	122.34 (12)
C3—C2—C10	110.25 (10)	O2—C9—C10	121.57 (12)
O1—C2—C1	102.26 (10)	N2—C9—C10	115.97 (11)
C3—C2—C1	109.79 (11)	C9—C10—C2	115.66 (11)
C10—C2—C1	110.02 (11)	C9—C10—H10A	108.4
C4—C3—C8	118.34 (12)	C2—C10—H10A	108.4
C4—C3—C2	121.13 (11)	C9—C10—H10B	108.4
C8—C3—C2	120.52 (11)	C2—C10—H10B	108.4
C5—C4—C3	121.46 (11)	H10A—C10—H10B	107.4
C5—C4—H4	119.3	C6—N1—H1A	115.2 (13)
C3—C4—H4	119.3	C6—N1—H2A	112.7 (13)
C4—C5—C6	120.04 (12)	H1A—N1—H2A	112.4 (18)
C4—C5—H5	120.0	C9—N2—C8	124.10 (11)
C6—C5—H5	120.0	C9—N2—H1B	115.3 (12)
C7—C6—C5	119.40 (11)	C8—N2—H1B	120.3 (12)
C7—C6—N1	121.48 (11)	C2—O1—H1C	111.2 (14)
			
F1—C1—C2—O1	64.75 (14)	C4—C5—C6—N1	−175.69 (13)
F2—C1—C2—O1	−174.61 (13)	C5—C6—C7—C8	−0.3 (2)
F3—C1—C2—O1	−54.94 (14)	N1—C6—C7—C8	176.28 (12)
F1—C1—C2—C3	−56.07 (14)	C6—C7—C8—C3	−1.0 (2)
F2—C1—C2—C3	64.58 (15)	C6—C7—C8—N2	176.92 (12)
F3—C1—C2—C3	−175.76 (11)	C4—C3—C8—C7	1.62 (19)
F1—C1—C2—C10	−177.58 (11)	C2—C3—C8—C7	−179.61 (12)
F2—C1—C2—C10	−56.93 (16)	C4—C3—C8—N2	−176.26 (12)
F3—C1—C2—C10	62.73 (14)	C2—C3—C8—N2	2.50 (19)
O1—C2—C3—C4	−34.07 (17)	O2—C9—C10—C2	−147.86 (14)
C10—C2—C3—C4	−158.94 (12)	N2—C9—C10—C2	36.06 (18)
C1—C2—C3—C4	79.68 (15)	O1—C2—C10—C9	−167.21 (11)
O1—C2—C3—C8	147.20 (12)	C3—C2—C10—C9	−40.75 (16)
C10—C2—C3—C8	22.32 (16)	C1—C2—C10—C9	80.49 (15)
C1—C2—C3—C8	−99.05 (14)	O2—C9—N2—C8	174.33 (13)
C8—C3—C4—C5	−0.9 (2)	C10—C9—N2—C8	−9.6 (2)
C2—C3—C4—C5	−179.71 (13)	C7—C8—N2—C9	171.88 (13)
C3—C4—C5—C6	−0.4 (2)	C3—C8—N2—C9	−10.2 (2)
C4—C5—C6—C7	1.0 (2)		

### Hydrogen-bond geometry (Å, °)

**Table:**

*D*—H···*A*	*D*—H	H···*A*	*D*···*A*	*D*—H···*A*
N1—H1A···O1^i^	0.91 (2)	2.17 (2)	3.0409 (16)	160.0 (18)
N2—H1B···O2^ii^	0.92 (2)	1.99 (2)	2.9075 (15)	175.3 (18)
O1—H1C···N1^iii^	0.82 (2)	2.02 (2)	2.8280 (16)	168 (2)
N1—H2A···F3^iv^	0.87 (2)	2.41 (2)	3.1124 (16)	138.5 (17)
C4—H4···F2^v^	0.93	2.31	3.1846 (16)	156.

Symmetry codes: (i) *x*+1, *y*, *z*; (ii) −*x*+1, −*y*, −*z*; (iii) −*x*+1, −*y*, −*z*+1; (iv) *x*+1, −*y*+1/2, *z*+1/2; (v) *x*, −*y*+1/2, *z*+1/2.
